# Seasonal Variation of Polyphenols and Pigments in Ginkgo (*Ginkgo biloba* L.) Leaves: Focus on 3′,8″-Biflavones

**DOI:** 10.3390/plants13213044

**Published:** 2024-10-30

**Authors:** Iva Jurčević Šangut, Dunja Šamec

**Affiliations:** Department of Food Technology, University North, Trg dr. Žarka Dolinara 1, 48000 Koprivnica, Croatia; ijurcevic@unin.hr

**Keywords:** biflavonoids, *Ginkgo biloba* L., leaves, phenolic compounds

## Abstract

Ginkgo (*Ginkgo biloba* L.) is a widely recognized medicinal plant, often grown as an ornamental species in parks around the world. Its leaves change color from green in spring to yellow in autumn. In this study, we collected ginkgo leaves at seven developmental stages from May to November and measured chlorophylls, carotenoids, flavonoids, and antioxidant activity. The total polyphenol content showed a significant increase from May to November, rising from 15.15 ± 0.14 mg GAE g^−1^ dw to 45.18 ± 0.42 mg GAE g^−1^ dw. The total flavonoid content reached its peak in August at 5.87 ± 0.18 mg GAE g^−1^ dw. In contrast, the highest concentrations of total polyphenolic acids (4.13 ± 0.16 mg CAE g^−1^ dw) and antioxidant activity (306.95 ± 3.47 µmol TE g^−1^) were recorded in May. We specifically focused on a less-studied group of dimeric flavonoids or biflavonoids—3′,8″-biflavones. We identified five 3′,8″-biflavones (amentoflavone, bilobetin, ginkgetin, isoginkgetin, and sciadopitysin) throughout all developmental stages. Sciadopitysin was the most abundant biflavonoid, with its concentration rising from 614.71 ± 5.49 µg g^−1^ dw in May to 2642.82 ± 47.47 µg g^−1^ dw in November. Alongside sciadopitysin, the content of other biflavonoids (excluding amentoflavone) generally increased over the same period. This trend is further highlighted by the total biflavonoid content, which grew from 1448.97 ± 6.63 µg g^−1^ dw in May to 6071.67 ± 97.15 µg g^−1^ dw in November. We observed a negative correlation between biflavonoid and chlorophyll content, which may indicate their involvement in leaf senescence. However, this hypothesis warrants further investigation.

## 1. Introduction

Flavonoids are diverse group of specialized plant metabolites. Their chemical structure is based on a C6-C3-C6 skeleton, consisting of two benzene rings (rings A and B) connected by a 3-carbon chain, which often forms a third ring (ring C). Due to structural variations, flavonoids are classified into several categories, including flavanones, flavanols, flavones, and flavonols. Other classes of flavonoid compounds include isoflavones, biflavonoids, flavonolignans, prenylflavonoids, flavonoid glycoside esters, aurones, and chalcones [1]. Structural modifications can vary and include processes such as hydroxylation, methylation, and glycosylation. All these structural features of flavonoids may influence their biological activities [2]. Flavonoids, despite being known as secondary metabolites of plants, play a significant role in various plant functions, particularly in the context of plant–environment interactions and a more appropriate name for them would be specialized metabolites. In plants, they serve as defences against pathogenic insects and fungi, act as antioxidants, and function as antimicrobial agents [3]. Flavonoids are widely recognized as molecules with significant biological activity and health-promoting properties [1].

Ginkgo (*Ginkgo biloba* L.) is a medicinal plant, known also as a living fossil and the only surviving member of the Ginkgoaceae family. The unique botanical features of ginkgo are most evident in its fan-shaped leaves with dichotomous venation, which, along with their seasonal colour changes, contribute to the ornamental value of this species. Although this species originates from China, it is now widespread across the globe. Ginkgo is characterised by its remarkable resilience to various abiotic and biotic stresses. A testament to this resilience is the fact that several specimens survived the nuclear bombing of Hiroshima [4]. Specialized metabolites are considered one of the key factors contributing to ginkgo’s excellent adaptation to various environmental conditions [5]. Ginkgo has long been recognised in traditional medicine as a source of phytochemicals beneficial for health and is often mentioned in the context of cardiovascular diseases, cancer, cognitive dysfunctions, asthma, bronchitis, and more [4]. The health benefits of ginkgo are attributed to its rich composition of bioactive components such as flavonoids, terpenes, and trilactones [6]. The European Pharmacopoeia proposed that the standard leaf extract contains 5–7% terpene lactones and 22–27% flavone glycosides [7]. According to the review by Liu et al. [6], 110 flavonoids have been detected in ginkgo, most commonly in the form of aglycones, glycosides, and biflavonoids.

Biflavonoids, dimeric forms of flavonoids, consist of either two identical or different flavonoid units and most commonly form combinations such as flavone–flavone, flavone–flavanone, and flavanone–flavanone. Biflavonoids are particularly associated with plants used in both traditional and modern medicine [3]. Dimerization influences their biological activity [8], positioning them as a key focus for research and the potential development of new pharmaceuticals. According to He et al. [9], approximately 600 different biflavonoids have been reported, which are divided into 24 subgroups based on the position of the C-C linkage. To date, 13 biflavonoids have been identified in ginkgo, with the most common being amentoflavone, bilobetin, ginkgetin, isoginkgetin, and sciadopitysin [3]. The biflavonoids found in ginkgo belong to the flavone group, which forms a bond at the 3’C–8″C position and are therefore known as 3′,8″-biflavones. They are recognized as compounds with potential pharmacological applications in the treatment of various cancers, neurological and cardiovascular disorders, and as antiviral agents [3,10,11,12]. The highest concentrations of 3′,8″-biflavones have been recorded in the leaves of ginkgo, as well as in other parts of the plant directly exposed to the environment, such as bark, petioles, and seed sarcotesta [13]. Given their localisation, they may play a role in protecting against pathogens and mitigating the effects of UV radiation, but their exact role in plants remain unknown [13].

Flavonoids serve as biomarkers of the external environment, with factors such as light, drought, temperature, and nutrient availability playing a crucial role in their synthesis and accumulation in ginkgo leaves [14]. Consequently, the timing of leaf harvesting naturally exerts a significant influence on the composition of phytochemicals [15,16,17,18,19,20]. Previous research on ginkgo has primarily focused on variations in flavonoid glycosides, including quercetin, kaempferol, and isorhamnetin, with the goal of identifying differences in their content between green and yellow leaves [19,21]. Kobus-Cisowska et al. [21] associate the increased content of flavonoid compounds with the higher antioxidant activity of autumn extracts. Similar results were published by Zheng et al. [19], who compared methanol extracts of ginkgo leaves collected in June and October. Comparative metabolomic and transcriptomic studies suggest a potential role for flavonoid compounds in the regulation of leaf senescence [22,23].

However, data on biflavonoids, particularly 3′,8″-biflavones, are limited, with no studies to date reporting their seasonal variation. Therefore, this study aims to investigate the seasonal variation of individual biflavonoids in ginkgo leaves. We profiled the distribution of the five most prevalent 3′,8″-biflavones—amentoflavone, bilobetin, ginkgetin, isoginkgetin, and sciadopitysin—in ginkgo leaves throughout the entire growing season, from May to November (seven development stages). We also correlated their content with pigments, total polyphenols, flavonoids, phenolic acids, and antioxidant activity (using the DPPH radical assay).

## 2. Results and Discussion

### 2.1. Seasonal Variation of Pigments in Ginkgo Leaves

**Figure 1:**
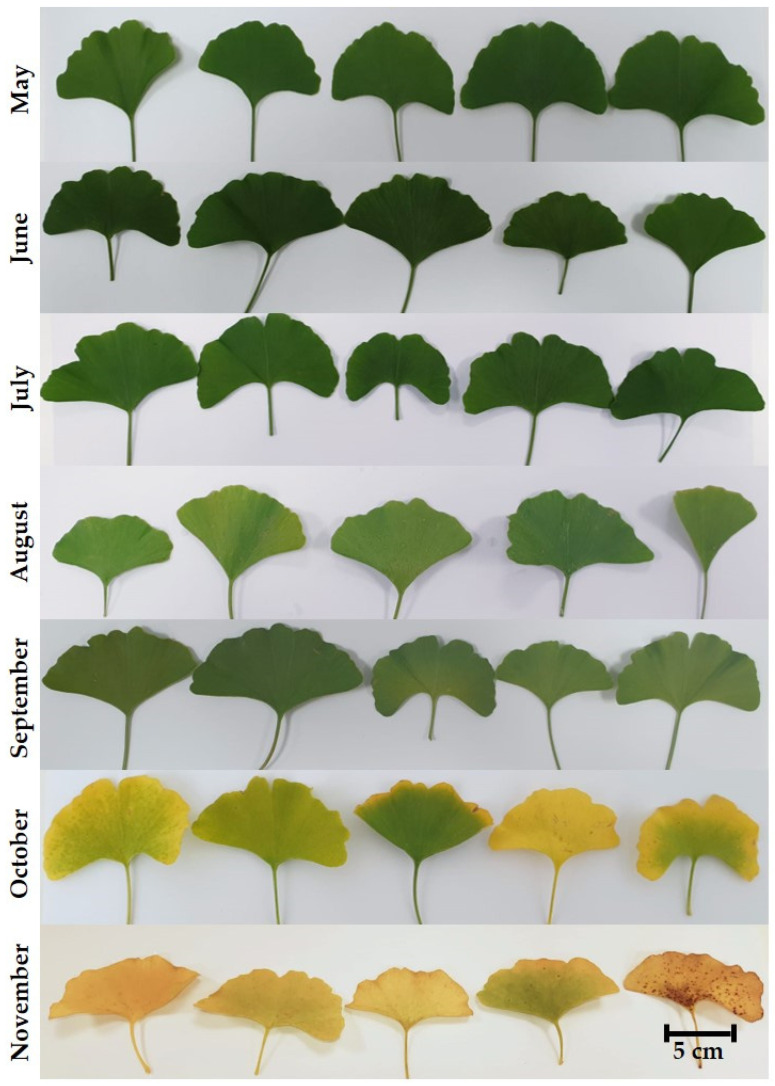
Ginkgo leaves during the 2022 growing season from May, June, July, August, September, October, and November (from top to bottom).

Throughout the growing season, we monitored the change in leaf color. As part of a normal physiological process, leaves change color from green in May to yellow in October, as shown in Figure 1.

Changes in leaf color are linked to variations in pigment content, so we measured the levels of photosynthetic pigments. The levels of chlorophyll *a* and *b*, as well as total chlorophylls, total carotenoids, and the ratios of chlorophyll a to b and chlorophylls to carotenoids throughout the growing season of ginkgo leaves are shown in Table 1.

The levels of chlorophyll *a*, chlorophyll *b*, and total chlorophyll in ginkgo leaves follow a similar trend throughout the growing season. Both types of chlorophyll steadily increase from May, peaking in July with concentrations of 1.71 ± 0.06 µg g^−1^ dw for chlorophyll *a* and 0.35 ± 0.01 µg g^−1^ dw for chlorophyll *b*. After reaching these maximum levels, the chlorophyll concentrations gradually decrease until the end of the growing season. The ratio of chlorophyll *a* to *b* remains stable throughout most of the growing season, but shows a significant decrease in the autumn leaf samples from October and November. A decrease in the chlorophyll *a*/*b* ratio occurs during leaf senescence. During this process, the chlorophyll *a* degrades more rapidly than chlorophyll *b*, leading to a relative increase in the proportion of chlorophyll *b*. This change can be associated with the enlargement or restructuring of the antenna system of Photosystem II (PS II) as the plant prepares for the end of the growing season [24]. The trend of decreasing chlorophyll content during senescence has already been observed in ginkgo trees. Zhang et al. [25] and Li et al. [22] reported that chlorophyll levels in ginkgo leaves begin to decline in early September, signalling the onset of autumn. This observation is consistent with our results.

The total carotenoid content, wh exhibited greater variation throughout the growing season. It increased from May to July, peaking during this period. This was followed by a significant decline in concentration from August to September, after which the levels rose again towards the end of the growing season. This trend is expected, as the yellow color of autumn leaves is traditionally associated with carotenoids. As chlorophyll degrades, the green pigment in the leaves diminishes, leading to the gradual accumulation of significant amounts of carotenoids and a color change [26]. The significant drop in chlorophyll observed in October may accelerate leaf senescence. However, since carotenoids did not dominate over chlorophyll, the yellowing of ginkgo leaves cannot be attributed to carotenoids alone. Other pigments, such as flavonoids, may also contribute to the yellow coloration. The mechanism underlying leaf coloration in ginkgo remains poorly understood and it may be that flavonoids are associated with the yellow coloration in addition to carotenoids [23].

### 2.2. Seasonal Variation of Total Polyphenols, Total Flavonoids, and Total Phenolic Acids in Ginkgo Leaves

**Figure 2:**
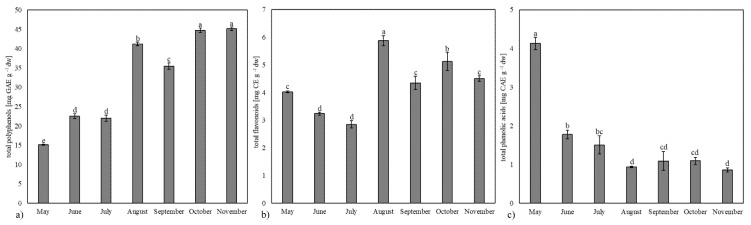
Total polyphenols (**a**), flavonoids (**b**), and phenolic acids (**c**) in ginkgo leaves during the growing season. Values marked with different letters are significantly different at *p* < 0.05.

Polyphenolic compounds may also contribute to the leaf’s coloration [27]. Thus, we determinate the total polyphenols, flavonoids, and phenolic acids content during the growing season (Figure 2).

The total polyphenol content increases from the beginning of the season until August, after which there is a decline in concentration. The highest value of polyphenols was recorded in leaf samples collected at the end of the growing season, in October (44.75 ± 0.55 mg GAE g^−1^ dw) and November (45.18 ± 0.42 mg GAE g^−1^ dw). In the case of flavonoids, the highest content was observed in the leaves collected in August (5.87 ± 0.18 mg CE g^−1^ dw), followed by a notable presence in the autumn leaves from October, as well as in September and November. These results are consistent with studies suggesting that increased flavonoid synthesis in ginkgo is associated with higher levels of UV-B radiation [5,28,29]. In ginkgo, the expression pattern of genes related to flavonoid biosynthesis is mainly driven by environment [5]. Zhao et al. [30] exposed ginkgo seedlings to UV-B radiation, and observed increased expression of FLS and F3’H and increased content of quercetin, kaempferol, and isorhamnetin. Xu et al. [29] analyzed flavonols (total flavonol, quercetin, kaempferol, and isorhamnetin) content, flavonol yield per plant, and expression of flavonoid biosynthesis-related genes in 2-year ginkgo at four different light intensities (100, 76, 40, and 25% of full sunlight). The 100% sunlight produced the highest flavonoid content, which is with line with our results, which show the highest content of flavonoids in samples collected during the month when the sunlight is the strongest. Consistent with our findings, Rimkiene et al. [17] recommended July and August as the optimal months for harvesting ginkgo leaves to maximize the yield of flavonoids such as quercetin, kaempferol, and isorhamnetin. According to the review article by Mao et al. [31], the role of flavonoids in ginkgo is closely related to environmental conditions, particularly in protecting the plant from UV-B radiation, drought stress, and biotic stress, including antiviral and antifungal activities. Their synthesis is regulated by several transcription factors, including MYB, bZIP, bHLH, WD40, NAC, and TCP. Additionally, hormones, miRNAs, and lncRNAs also play a role in regulating flavonoid synthesis. The review notes that the flavonoid content in the leaves of young trees is significantly higher than that in adult trees, indicating that only the leaves of young trees are suitable as raw materials for drug production, thereby highlighting the importance of tree age in flavonoid accumulation. However, our findings, which align with other studies [17,28,29], suggest that certain flavonoids may also increase during later developmental stages. According to our results, only phenolic acids were significantly higher in May (4.13 ± 0.16 CAE mg^−1^ dw), followed by a decline in their content in the subsequent months. These results are similar to studies on free and bound phenolic acids in leaves of *Centaurea* sp. [32] and walnuts (*Juglans regia* L.) [33], where the highest content of phenolic acids was recorded at the beginning of the growing season.

### 2.3. Seasonal Variation of 3′–8″ Biflavones in Ginkgo Leaves

**Figure 3:**
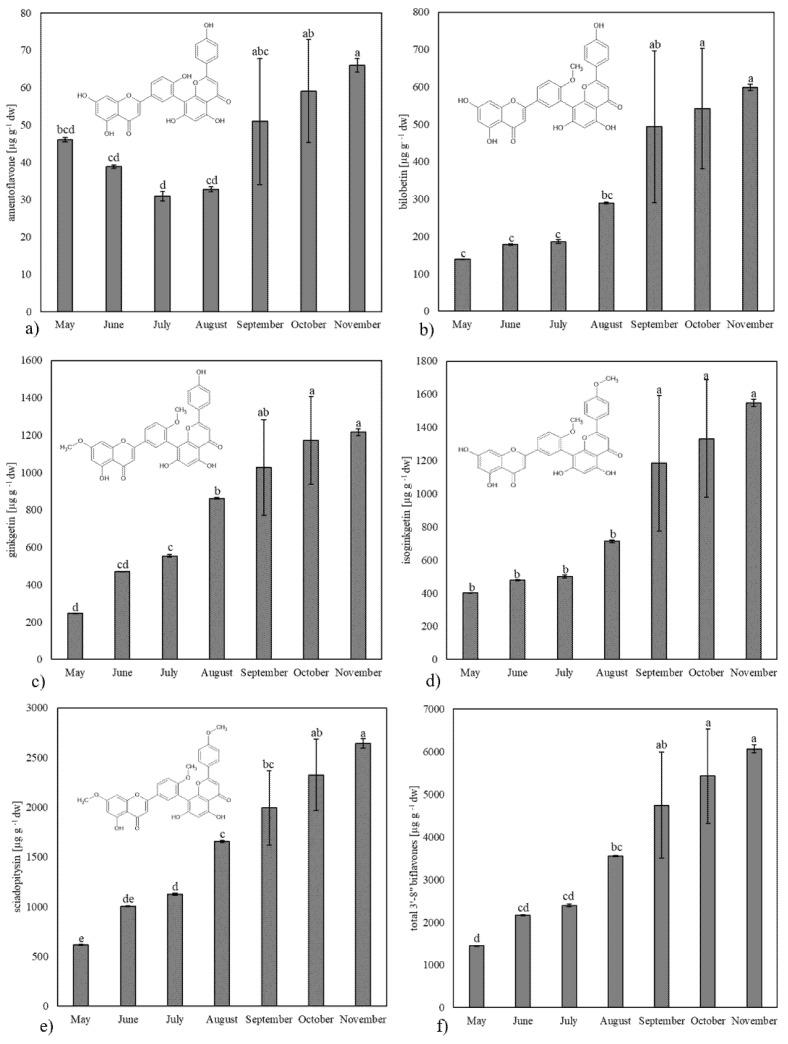
The content of biflavonoids during the season: amentoflavone (**a**), bilobetin (**b**), ginkgetin (**c**), isoginkgetin (**d**), sciadopitysin (**e**), and total 3′,8″-biflavones (**f**) in ginkgo leaves during the growing season. Values marked with different letters are significantly different at *p* < 0.05.

As mentioned above, the color of yellow leaves may be due to the presence of flavonoids, which have a yellow color. In 1929, the yellow pigment was isolated from yellow gingko leaves [34], and identified as ginkgetin, a dimeric flavonoid which belongs to the group of 3′,8″-biflavones [35]. Later, isoginkgetin, biolobetin, sciadopitysin and amentoflavone were also reported in ginkgo leaves as a main 3′,8″-biflavones [13,36,37,38]. The content of those five main biflavones as well the total biflavones content during the growing season in ginkgo leaves are shown on Figure 3.

Throughout the growing season, the ratio of identified biflavones remains stable, with sciadopitysin being the most abundant, ranging in concentration from 614.74 to 2642.82 µg g^−1^ dw. This aligns with reports in the literature, which identify sciadopitysin as the most abundant biflavone in ginkgo leaves [13,38,39,40]. Biflavones named after ginkgo, such as isoginkgetin and ginkgetin, were detected in significantly lower amounts than sciadopitysin. Ginkgetin levels ranged from 247.61 to 1215.98 µg g^−1^ dw, while isoginkgetin concentrations varied between 401.35 and 1547.95 µg g^−1^ dw. Amentoflavone was the least prevalent among the biflavonoids, with concentrations ranging from 30.87 to 65.96 µg g^−1^ dw, followed by bilobetin, which ranged from 139.23 to 598.96 µg g^−1^ dw. According to the literature, geolocation [16] and the age [41] of the tree may influence biflavonoid composition. However, our data indicate that the ratio of biflavonoids remains consistent throughout the growing season. Notably, we observed an increasing trend in the total biflavonoid content from May to November for all biflavonoids except amentoflavone, which followed a distinct pattern. Studies on the seasonal variation of biflavonoids in ginkgo and other plants are quite limited. Yang et al. [42] examined the seasonal variation of the biflavonoids ginkgetin and amentoflavone in the needles of Chinese yew (*Taxus wallichiana* var. *maire*). The highest concentration of ginkgetin was observed in needles collected in August, while amentoflavone levels fluctuated throughout the season without a clear increasing or decreasing trend. The authors found no correlation between flavonoid accumulation and environmental factors such as temperature, daylight length, relative humidity, or rainfall. Wang et al. [39] reported the levels of bilobetin, ginkgetin, isoginkgetin, and sciadopitysin at six developmental stages of yellowing ginkgo leaves collected from mid-October to mid-November. Only sciadopitysin showed higher concentrations at the later stages, while the other biflavonoids exhibited significant fluctuations, with some not being detected just in some developmental stages. The study lacked precise details on leaf collection protocols and biological replicates, which may have affected the reliability of the results. Our data show that, in the later developmental stages, there is a larger standard deviation and greater variability in results between trees compared to younger leaves. Accurate leaf sampling is therefore critical for tracking the dynamics of biflavonoid accumulation effectively.

### 2.4. Seasonal Variation of Antioxidant Activity in Ginkgo Leaves

**Figure 4:**
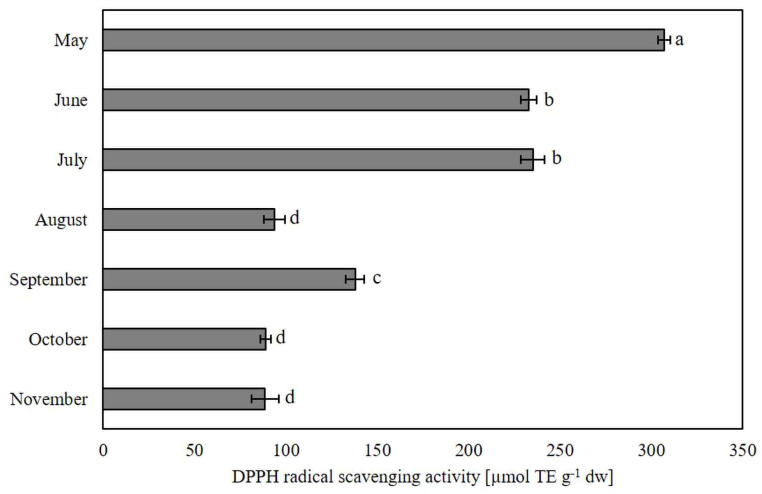
Antioxidant activity measured by DPPH in ginkgo extracts prepared from leaves collected during the growing season. Values marked with different letters are significantly different at *p* < 0.05.

As we have previously shown, the composition of phytochemicals in ginkgo leaf extract varies throughout the season. Consequently, the ability to scavenge free radicals also changes during different growth periods. Antioxidant activity in ginkgo leaves throughout the growing season is illustrated in Figure 4.

The highest antioxidant activity was recorded in leaves harvested at the beginning of the season in May (306.95 ± 3.47 µmol TE g^−1^ dw), followed by a gradual decline until August (93.75 ± 5.85 µmol TE g^−1^ dw). A significant increase in antioxidant activity was observed in the September samples (137.91 ± 4.96 µmol TE g^−1^ dw), while the levels in leaves collected at the end of the growing season were comparable to those measured in August. These results contrast with the findings of Kobus-Cisowska et al. [21], who reported higher antioxidant activity in ginkgo leaf extracts from yellow, autumn leaves in October compared to summer leaves from August, as determined by DPPH free radical assays. The difference was attributed to the increased content of flavonoids such as quercetin, rutin, myricetin, morin, isorhamnetin, and kaempferol, as well as total phytosterols. Our observed trend in antioxidant activity correlates with the dynamics of phenolic acids, which are notably higher in the first part of the growing season. Although levels of other phenolic compounds, including 3′,8″-biflavones, increase from August onwards, these biflavones have not demonstrated significant antioxidant activity in *in vitro* systems [8].

### 2.5. Correlation Analysis of Measured Parameters

To determine the correlations between the measured parameters, we created a correlation matrix, as shown in Table 2.

A strong positive correlation (blue) was observed between green pigments, and between both individual and total biflavonoids, as well as between total polyphenols and biflavonoid levels. This confirms that biflavonoids are a significant group of polyphenols in ginkgo leaves, whose seasonal increase contributes to the overall rise in total polyphenols. Despite being less studied than other polyphenols [3], biflavonoids play a key role in ginkgo. Interestingly, compared to other flavonoids, biflavonoids exhibit weaker antioxidant activity, as recently reported [8]. This is further supported by the correlation matrix, which shows a negative correlation (red) between DPPH antioxidant activity and both individual and total biflavonoid content. At growth stages where biflavonoid accumulation was higher, antioxidant activity was lower. Antioxidant activity, as measured by DPPH, was instead positively correlated with total phenolic acid content, with both parameters peaking early in the season.

A negative correlation was observed between total and individual biflavonoids and chlorophyll *a*, chlorophyll *b*, and total chlorophylls. This suggests that higher chlorophyll content (as seen in Table 1 and Figure 3) is associated with lower biflavonoid levels, possibly indicating a role for biflavonoids in leaf senescence. Although the exact function of biflavonoids in plants remains unclear, there are some indications about their involvement in photosynthesis inhibition. For instance, a study using spinach chloroplasts demonstrated that biflavonoids isolated from *Selaginella lepidophylla* inhibit ATP synthesis and other key photosynthetic processes, including electron transport, Photosystem II (PSII), Photosystem I (PSI), and their partial reactions within chloroplasts [43]. Additionally, the localization of biflavonoids in plant tissues exposed to the external environment [13] and in the outer regions of photosynthetic organs [44,45] may suggest their potential role in photosynthesis, though these hypotheses require further confirmation.

## 3. Materials and Methods

### 3.1. Chemicals, Reagents, and Instruments

To prepare the ginkgo extracts, we used 96% ethanol obtained from GRAM-MOL (Zagreb, Croatia). The following chemicals were used for phenolic component analysis: gallic acid (98%, Acros Organics, China), Folin–Ciocalteu reagent (Sigma Aldrich, Buchs, Switzerland), sodium carbonate (T.T.T., Sveta Nedelja, Croatia), (+)-catechin hydrate (Sigma Aldrich, St. Louis, MO, USA), aluminum chloride hexahydrate (Thermo Fischer Scientific, Kandel, Germany), hydrochloric acid (36.5%, Kemika, Zagreb, Croatia), sodium hydroxide (T.T.T., Sveta Nedelja, Croatia), sodium nitrite (Kemika, Zagreb, Croatia), sodium molybdate (VI) dihydrate (Sigma Aldrich, St. Louis, MO, USA), and caffeic acid (≥98%, HPLC grade, Sigma Aldrich, St. Louis, MO, USA).

All biflavonoid standards, including amentoflavone, ginkgetin, isoginkgetin, bilobetin, and sciadopitysin, were procured from PhytoLab (Vestenbergsgreuth, Germany, HPLC grade). Acetonitrile (VWR Chemicals, Radnor, PA, USA), formic acid (98–100%, Sigma Aldrich, Darmstadt, Germany), and ultrapure water (ELGA LabWater, Wycombe, UK) were used to prepare the mobile phases. For measuring antioxidant activity, we used 2,2-diphenyl-1-picrylhydrazyl (DPPH, Sigma Aldrich, Steinheim, Germany), methanol (Kemika, Zagreb, Croatia), and (±)-6-hydroxy-2,5,7,8-tetramethylchromane-2-carboxylic acid (97%, Sigma Aldrich, Buchs, Switzerland). Pigment determination was carried out using an acetone extract prepared with acetone from GRAM-MOL (Zagreb, Croatia).

The ginkgo leaves were dried using a freeze dryer (LIO-5PLT, KAMBIČ, Ljubljana, Slovenia), and then ground into a fine powder using a bead mill (Bead Ruptor 12, Omni International, Kennesaw, GA, USA). Ginkgo powder samples and reagent quantities for chemical preparations were accurately weighed on an analytical balance (Adam Equipment, Maidstone, UK). The extraction process was facilitated by an ultrasonic bath, a mechanical rotator (Bio RS-24, Biosan, Riga, Latvia), and a vortex mixer (V-1 plus, Biosan, Riga, Latvia). Supernatant separation was achieved via centrifugation (LMC-4200R, Biosan, Riga, Latvia). Spectrophotometric analyses were performed using a UV-VIS spectrophotometer (ONDA TOUCH UV-21, China), while biflavonoid quantification was carried out with an Agilent 1260 Infinity II high-performance liquid chromatography (HPLC) system (Agilent, Santa Clara, CA, USA) equipped with a diode array detector (DAD).

### 3.2. Plant Material and Sample Preparation

Ginkgo leaves were collected monthly throughout the growing season (from May to November) in 2022 from an alley of five ginkgo trees (46°09′20″ N; 16°49′46″ E) in an urban area in Koprivnica, Croatia. All trees were 30 years old, had a height of around 8 m, and were uniformly spaced 6–8 m apart. To ensure consistent leaf collection, leaves were gathered from each tree at a height of 2 to 2.5 m above the ground, with similar collection methods applied across all five tree sites. Each ginkgo tree represented a separate biological replicate. After collection, the leaves were immediately stored in a freezer at −80 °C until lyophilization. The lyophilization process was carried out for 48 h in a freeze dryer at approximately −102 °C and under pressures up to 0.3303 mBar. Once lyophilized, the leaves were ground into a fine powder using a bead mill, and this powder was then used for subsequent analyses.

### 3.3. Determination of Pigments

Chlorophyll pigments and carotenoids in the ginkgo leaf extract were quantified using the method described by Lichtenthaler and Buschmann [46]. Ten milligrams of the powdered sample were mixed with one milliliter of pure acetone, then centrifuged to separate the supernatant. Absorbance measurements of the extract were taken at three wavelengths (661.6 nm, 644.8 nm, and 470 nm). Specific equations, based on the solvent used, were applied to determine the concentrations of the pigments. All measurements were performed in triplicate, and results are expressed in mg per gram of dry weight (dw).

### 3.4. Determination of Total Polyphenols, Total Flavonoids, and Phenolic Acids

The preparation of ginkgo extract for determining total polyphenols, flavonoids, phenolic acids, and biflavonoids was conducted as follows: sixty milligrams of the powdered sample were mixed with two millilitres of 70% ethanol. The mixture was briefly vortexed and then subjected to ultrasonic treatment for 10 min at room temperature. Following this, the samples were placed on a rotary mixer for 45 min. Finally, the samples were centrifuged at 4000 RPM for 10 min, and the supernatant was collected for further analysis.

Measurements were conducted as previously reported [36]. Total polyphenols were determined using the Folin–Ciocalteu method [47], total flavonoids were quantified using the modified colorimetric method of Zhishen et al. [48], and total phenolic acids were measured using Arnow’s reagent method [49]. All analyses were performed in triplicate. The concentrations of phenolic compounds in the samples were calculated from standard curves and expressed as µg mg^−1^ of dry weight (dw).

### 3.5. Determination of 3′,8″-Biflavones with HPLC-DAD

To determine the 3′,8″-biflavones in ginkgo leaves (amentoflavone, bilobetin, ginkgetin, isoginkgetin, and sciadopitysin), we employed a slightly modified method based on Kovač Tomas et al. [13], using an Agilent 1260 Infinity II high-performance liquid chromatography (HPLC) system (Agilent, Santa Clara, CA, USA) with a diode array detector (DAD). First, standards were prepared as stock solutions at a concentration of 1 mg/mL in pure DMSO, then diluted with methanol to create working solutions at concentrations of 1, 10, 50, and 100 µg mL^−1^. Each concentration was prepared in triplicate. Compound separation was achieved using a Zorbax 300Extend-C18 column (Agilent, Santa Clara, CA, USA) at 40 °C. The mobile phases were 0.1% formic acid solution (mobile phase A) and acetonitrile (mobile phase B), with a flow rate of 1 mL min^−1^ over a 45 min analysis period. The gradient elution profile was as follows: 0 min 98% A, 10 min 79% A, 15 min 77% A, 20 min 75% A, 25 min 64% A, 30 min 62% A, 35 min 51% A, 40 min 25% A, 43 min 8% A, and 45 min 98% A. Sample and standard injections were 10 µL each. Before injection, samples were filtered through a 45 µm pore size polytetrafluoroethylene syringe filter. Data processing was performed using Agilent OpenLab CDS software (version 2.6). Biflavone identification was achieved by comparing sample spectra with those of the standards. Chromatograms were recorded at 330 nm. The concentrations of 3′–8″ biflovones in the samples were calculated from standard curves and expressed as µg mg^−1^ of dry weight (dw).

### 3.6. Antioxidant Activity

Antioxidant activity was assessed using the modified Brand-Williams method [50] using the DPPH (1,1-diphenyl-2-picrylhydrazyl), as we reported previously [34]. A total of 5 µL of 70% ethanolic extract was mixed with 980 µL of 0.094 mM methanolic DPPH solution. After one hour, the absorbance was measured at 515 nm, and the results were expressed as Trolox Equivalents per gram of dry weight (µmol TE g^−1^ dw).

### 3.7. Statistical Analysis

The sampling of leaves was conducted from five ginkgo trees which represent five biological replicates. All measurements were performed at least in triplicate, and results are presented as the mean value ± standard deviation (SD). Statistical data analysis was conducted using PAST software (version 4.15), employing one-way ANOVA and post hoc Tukey’s test. Differences between samples were considered significant at *p* < 0.05

## 4. Conclusions

We collected ginkgo leaves at seven developmental stages throughout the season, from May to November, and assessed chlorophyll content, carotenoids, total polyphenols, total flavonoids, total phenolic acids, and antioxidant activity spectrophotometry. Additionally, we measured the content of 3′,8″-biflavones using HPLC-DAD. Throughout the season, we observed increases in total carotenoid content, total polyphenols, flavonoids, and total 3′,8″-biflavones, including individual biflavonoids such as bilobetin, ginkgetin, isoginkgetin, and sciadopitysin. Amentoflavone was the least abundant and did not increase over the season. Sciadopitysin was the most abundant biflavonoid at all stages. We also detected a negative correlation between biflavonoid and chlorophyll content, suggesting that biflavonoids may be involved in leaf senescence. This potential role warrants further investigation.

## Figures and Tables

**Table 1:** Monthly variation of pigments (chlorophyll *a*, chlorophyll *b*, total chlorophylls, total carotenoids, and the ratios of chlorophyll *a* to *b* and chlorophylls to carotenoids) in ginkgo leaves throughout the growing season. Values marked with different letters are significantly different at *p* < 0.05.

Season 2020	Chlorophyll *a* (mg g ^−1^ dw)	Chlorophyll *b* (mg g^−1^ dw)	Total Chlorophylls (mg g^−1^ dw)	Total Carotenoids (mg g^−1^ dw)	Chlorophyll *a*/Chlorophyll *b*	Chlorophylls/ Carotenoids
May	0.68 ± 0.04 ^c^	0.15 ± 0.04 ^bc^	0.84 ± 0.08 ^c^	0.02 ± 0.00 ^e^	4.73 ± 0.91 ^a^	56.65 ± 18.03 ^a^
June	0.96 ± 0.04 ^b^	0.18 ± 0.02 ^b^	1.13 ± 0.06 ^b^	0.03 ± 0.00 ^de^	5.42 ± 0.29 ^a^	34.09 ± 0.65 ^ab^
July	1.71 ± 0.06 ^a^	0.35 ± 0.01 ^a^	2.07 ± 0.07 ^a^	0.24 ± 0.01 ^a^	4.83 ± 0.09 ^a^	8.57 ± 0.06 ^c^
August	0.87 ± 0.01 ^b^	0.17 ± 0.01 ^b^	1.04 ± 0.02 ^b^	0.05 ± 0.01 ^d^	5.08 ± 0.21 ^a^	20.35 ± 2.12 ^bc^
September	0.73 ± 0.01 ^c^	0.13 ± 0.0 ^bcd^	0.86 ± 0.01 ^c^	0.05 ± 0.00 ^d^	5.62 ± 0.17 ^a^	17.26 ± 0.61 ^bc^
October	0.20 ± 0.01 ^d^	0.09 ± 0.01 ^cd^	0.29 ± 0.02 ^d^	0.08 ± 0.00 ^c^	2.14 ± 0.21 ^b^	3.69 ± 0.39 ^c^
November	0.07 ± 0.00 ^e^	0.08 ± 0.01 ^d^	0.15 ± 0.01 ^d^	0.11 ± 0.01 ^b^	0.80 ± 0.08 ^c^	1.32 ± 0.16 ^c^

**Table 2:** Correlation matrix of measured parameters, in ginkgo leaves during season.

	chl a	chl b	Total chls	Total car	DPPH	TP	TF	TPA	a	b	g	i	s	TB
chl *a*	1													
chl *b*	0.961	1												
total chls	0.999	0.971	1											
total car	0.504	0.696	0.53	1										
DPPH	0.551	0.508	0.547	−0.016	1									
TP	−0.637	−0.576	−0.631	−0.007	−0.992	1								
TF	−0.592	−0.619	−0.598	−0.432	−0.730	0.744	1							
TPA	0.133	0.100	0.129	−0.334	0.851	−0.796	−0.330	1						
a	−0.899	−0.808	−0.890	−0.207	−0.464	0.556	0.289	−0.163	1					
b	−0.743	−0.669	−0.736	0.006	−0.840	0.877	0.496	−0.647	0.829	1				
g	−0.622	−0.551	−0.614	0.093	−0.948	0.960	0.581	−0.807	0.658	0.961	1			
i	−0.759	−0.671	−0.750	0.024	−0.822	0.865	0.470	−0.625	0.853	0.997	0.950	1		
s	−0.674	−0.584	−0.664	0.100	−0.921	0.945	0.541	−0.767	0.726	0.975	0.991	0.973	1	
TB	−0.702	−0.617	−0.693	0.067	−0.898	0.927	0.530	−0.728	0.766	0.990	0.987	0.987	0.997	1

Abbreviations: chlorophyll *a* (chl *a*), chlorophyll *b* (chl *b*), total chlorophylls (total chls), total carotenoids (total car), total polyphenols (TP), total flavonoids (TF), total phenolic acid (TPA), amentoflavone (a), bilobetin (b), ginkgetin (g), isoginkgetin (i), sciadopitisyn (s), total biflavones (TB).

## Data Availability

Data are contained within the article.
